# Approaching Cognitive Behavior Therapy For Generalized Anxiety Disorder From A Cognitive Process Perspective

**DOI:** 10.3389/fpsyt.2019.00796

**Published:** 2019-11-04

**Authors:** Colette R. Hirsch, Sarah Beale, Nick Grey, Sheena Liness

**Affiliations:** ^1^Department of Psychology, Institute of Psychology, Psychiatry and Neuroscience, King’s College London, London, United Kingdom; ^2^Centre for Anxiety Disorders and Trauma, South London and Maudsley NHS Foundation Trust, London, United Kingdom; ^3^Sussex Partnership NHS Foundation Trust, Worthing, United Kingdom

**Keywords:** generalized anxiety disorder, cognitive behavior therapy, attention bias, interpretation bias, verbal worry, attention control

## Abstract

Generalized anxiety disorder (GAD), with uncontrollable worry at its core, is a common psychological disorder with considerable individual and societal costs. Cognitive behavior therapy (CBT) is recommended as the first-line treatment for GAD; however, further investigation into its effectiveness in routine clinical care is indicated and improvement is required in treatment outcomes for worry. Improvements to CBT need to be guided by experimental research that identifies key mechanisms maintaining core aspects of the disorder. This paper summarizes how theory-driven experimental research guided selection and refinements of CBT techniques originally developed by Borkovec and Costello, to target key cognitive processes that maintain worry in GAD. Hirsch and Mathews’ model specifies three key research-supported processes that maintain uncontrollable worry in GAD: implicit cognitive biases such as negative interpretation bias and attention bias, generalized verbal thinking style, and impaired ability to re-direct attentional control away from worry. Specific CBT techniques outlined in this paper aim to target these key processes. Clinical data from clients treated using our refined CBT protocol for GAD in a routine clinical care service with a special interest in anxiety disorders were collected as part of service procedures. Large pre-to-posttreatment effect sizes were obtained for anxiety (GAD-7), depression (PHQ-9), and worry (PSWQ) (*d*=.90–2.54), and a moderate effect size was obtained for quality of life (WASA; *d*=.74). Recovery was indicated for 74% of cases for anxiety, 78% for depression, and 53% for worry. These findings exceeded most previous effectiveness studies in routine care and were in-line with GAD efficacy trials. This paper also outlines the application of specific clinical techniques selected, adapted or developed to target key cognitive mechanisms which maintain worry in GAD.

## Introduction

Generalized anxiety disorder (GAD) is a common and disabling condition with the hallmark symptom of persistent, excessive, and uncontrollable worry across a number of different topics (1). GAD has an estimated lifetime prevalence in European and American adults of 6–7% (2–5). If untreated, the disorder often persists chronically for decades and demonstrates high relapse rates if remission does occur (3, 6). Comorbidity with depression-spectrum disorders and other anxiety disorders, notably social anxiety disorder, specific phobia, and panic disorder, is common (4). GAD sufferers report disorder-related impairments in social functioning, occupational functioning, and overall quality of life (7, 8). GAD leads to societal costs associated with increased use of healthcare services and workplace absences (7, 9, 10), with estimated total costs (direct and indirect) of €5308 per patient per year in European samples (11) and estimated healthcare costs of $8613 per patient per year in North American samples (12, 13).

### Psychological Therapies For GAD

Given the high prevalence and considerable individual and societal burden of GAD, developing and disseminating efficient and effective interventions is essential. Receiving preferred treatment type impacts clients’ engagement and outcomes (14), and people with common mental health conditions, including GAD, exhibit a strong preference for psychological versus pharmacological treatments (15). Current guidelines recommend individual, face-to-face cognitive behavior therapy (CBT) as the first-line treatment for moderate-severe GAD (16–18). CBT refers to a range of interventions that aim to modify maladaptive cognitive processes, which are proposed to maintain psychological disorders such as GAD (19). While CBT was initially developed in the context of depression (20, 21), clinically useful GAD-specific CBT interventions have been developed and tested. A number of CBT protocols are recommended by NICE for use in the UK (e.g. 22–26).

Meta-analyses of randomized controlled trials of CBT for GAD (23, 27–29) consistently support its superiority for reducing anxiety and mood symptoms and improving quality of life post-treatment and long-term, compared to non-intervention and non-CBT control conditions. CBT trials demonstrate large effect sizes for the reduction of the core symptom of worry (30, 31). Unfortunately, the percentage of clients reaching standardized recovery criteria (32), i.e. a score of 47 or below on the Penn State Worry Questionnaire (PSWQ; 33), has been modest, with 46% achieving these criteria post-treatment and 57% at 12-month follow-up in gold-standard trials (31). Hence, despite encouraging results with large effects sizes evident, CBT trials have struggled to get patients below clinical cut-offs and into recovery on the key dimension of worry. To effectively address this core feature of GAD, understanding the cognitive factors that maintain pathological worry and using relevant evidence to inform interventions is important.

Additionally, the generalizability of outcomes from randomized control trials to routine clinical care has been questioned due to greater potential client complexity and lower motivation, as well as less time and resources (34), and therapist deviation from established protocols (35). A meta-analysis of the effectiveness of CBT for the treatment of adult anxiety disorders within routine care included 11 studies on GAD (36). CBT for GAD demonstrated large effects for pre-to-posttreatment reduction of anxiety and depression symptoms that were broadly in line with the comparison efficacy trials selected by the authors of the meta-analysis (22, 37, 38). However, outcomes in the meta-analysis (36) were based solely on generic measures of anxiety and depression, and did not specifically assess pre-to-posttreatment change on the core GAD symptom of worry, which is typically done using the PSWQ. Reliance on generic anxiety and depression questionnaires to assess clinical outcomes in GAD is common practice in routine care.

Given that worry is the defining symptom of GAD, selecting and refining CBT techniques that address the processes underlying pathological worry is essential. Consequently, further investigation of evidence-based CBT that selects interventions to target key processes that maintain worry, as well as anxiety, in GAD is indicated. The following evaluation focuses on disorder-specific and generic outcomes of a CBT intervention designed to target key cognitive-process maintaining pathological worry in GAD provided in a UK National Health Service (NHS) clinical service. First however, research into key processes underlying worry, the scientific basis for the CBT intervention, will be presented.

### Key Cognitive Processes Underlying Worry

Given that the central characteristic of GAD is uncontrollable worry, treatment needs to target key mechanisms that maintain worry. In keeping with treatment development for other disorders (e.g. social anxiety disorder; 39; posttraumatic stress disorder; 40), research needs to first identify processes that differentiate GAD and general populations. Subsequently, studies should confirm that the identified mechanism has a causal role in maintaining key aspects of the disorder, i.e. worry in the case of GAD.

Based on Hirsch and Mathews’ (41) theoretical model of pathological worry, three key candidate processes trigger and maintain bouts of worry: (i) automatic emotional-processing biases that lead to intrusions of negative thoughts into awareness, and then continue to operate during worry and generate thoughts about more negative potential outcomes, (ii) use of a generalized verbal thinking style during worry that prevents positive resolution and increases the likelihood of further episodes of worry, and (iii) impaired intentional control of attention that impedes terminating an episode of worry and re-focusing onto the task at hand or other non-worry topics.

#### Automatic Emotional-Processing Bias Favoring Negative Information

GAD is characterized by streams of thinking on wide-ranging topics imbued with emotional ambiguity about negative outcomes. Individuals with GAD exhibit automatic emotional-processing biases favoring negative information.

#### Interpretation Bias

People with GAD and other common mental health conditions tend to interpret ambiguous scenarios (e.g. “You wake with a start in the middle of the night, thinking you heard a noise, but all is quiet”) in a more threatening manner (e.g. “it’s a burglar”) than non-anxious control participants (e.g. “it was the wind”; 42–44). Participants with GAD are also more likely to produce threat-related spellings of homophones (words that sound the same but have two meanings e.g. dye/die) than non-anxious control participants. Interestingly, people in remission from GAD tend not to differ significantly from control groups (45, 46). Interpretation bias is also reduced following anxiolytic medication (46), with greater clinical improvement associated with correspondingly reduced negative interpretations. On a recognition memory test for ambiguous sentences, which assessed interpretation bias while avoiding assessor demand effects, participants with GAD endorsed more threatening interpretations than GAD-recovered and non-anxious participants, who performed similarly (47). The groups did not differ in their rejection of threatening but impossible interpretations (foils), thus ruling out the effects of a general threat-based response bias. Using the same task, Krahé et al. (48) further demonstrated that worry was associated with negative interpretation bias across the general population, and that participants with GAD were biased towards negative interpretations, while community volunteers were biased towards benign (i.e. neutral or positive) interpretations. Hirsch and Mathews' (41) model of worry posits a key role for negative interpretations in triggering negative thoughts and maintaining worry. In contrast, a bias to generate more benign interpretations, evident in non-anxious individuals, would lead to less worry being triggered by negative thoughts and to briefer bouts of worry when it occurs, due to benign interpretations having the potential to terminate streams of worry.

Given that there is a negative interpretation bias in individuals with GAD, the next question that must be addressed is whether it has a causal role in maintaining worry and anxiety. One effective approach to investigating causality is to isolate putative causal processes and modify them experimentally. A secondary benefit to this approach is determining how the mechanism can be modified to inform effective psychological intervention methods. Cognitive bias modification (CBM) of interpretation involves repeated practice with tasks that require generation of benign meanings for ambiguous events, or attending to benign interpretations while ignoring threatening meanings.

The causal role of negative interpretation bias in maintaining worry in GAD was initially established using single-session experiments in which participants were trained to generate benign interpretations of ambiguity. Hirsch et al. (49) showed that training high trait worriers to preferentially access benign meanings of emotionally ambiguous homographs (words with two meanings e.g. hit—record or hit—attack), and ambiguously threatening scenarios, led to reductions in worry. Positive training using the same approach was later demonstrated to be effective in reducing worry in participants with GAD on a behavioral task assessing intrusive thoughts, and showed that this effect was mediated by change in interpretive bias (50). These findings provided initial support for the causal role of interpretation bias in the maintenance of worry in GAD. To investigate the longer-term role of interpretation bias, Hirsch et al. (51) allocated volunteers with GAD to receive 10 home-based CBM sessions. CBM involved listening to either ambiguous worry-related scenarios where the ambiguity was resolved in benign ways, or to an active control condition where ambiguity was not resolved. The active training condition led to reduced negative interpretations post-training and, importantly, to reduced levels of trait worry, anxiety and depression at one-month follow-up. Hence, negative interpretation bias has a causal role in maintaining worry and anxiety in people with GAD in the longer term. Consequently, psychological interventions for GAD should focus on developing more benign interpretations of ambiguous situations.

#### Attention Bias

Another key emotional processing bias proposed in Hirsch and Mathews’ (41) model is selective attention to threatening information (attention bias), which heightens perceived threat in the environment, presumably leading to more worry. A typical paradigm to assess attention bias is the dot probe. This involves one threatening and one non-threatening word being presented briefly on screen and subsequently disappearing, with one word then replaced by a probe. Participants categorize the probe as quickly as possible and faster responses are assumed to indicate that the participant was attending to the location of the word that the target replaced. Numerous studies indicate that individuals with GAD preferentially attend to threatening information when simultaneously presented with both threatening and benign stimuli, including words (52–55) and faces (56, 57). However, some research using emotional face stimuli have failed to identify a negative attentional bias in GAD, and instead found faster shifting away of attention from negative faces in this population (58). Given that worry is a verbal process with infrequent imagery (59–61), verbal material may be more appropriate to assess attentional biases in people with GAD. Furthermore, attention bias may operate more clearly when worry has been primed. In high trait worriers, the tendency to attend to threat operated more strongly when tested after an episode of worry (62), creating a self-maintaining cycle in which worry itself may foster greater attention to threat, which in turn could perpetuate worry bouts. Hence, while an attentional bias to threat in GAD has been established, further research on attention biases in GAD is warranted to understand the boundaries (e.g. verbal material akin to worry) and setting conditions (e.g. once worry is activated) of attentional biases in this population.

Attentional bias does, however, appear to have a causal role in maintaining worry and anxiety in GAD. Cognitive bias modification of attention (CBM-A) has been used to test the causal role of attention bias in maintaining worry. Hayes, Hirsch, and Mathews (63) allocated high worriers to benign training or a control condition for a two-stage training session. Participants in the benign training group completed dot-probe training where a target probe replaced the non-threatening word nearly all the time, thus encouraging attention to non-threatening information. In contrast, the probe for the control group replaced threat and non-threat words equally often. Participants then completed dichotic listening training, where one worry story was played into one channel (ear) and one positive story was played into the other channel simultaneously via headphones. Participants were instructed to listen to a specified story, and at random points the story switched to the other ear so participants had to shift attention to the other channel. Participants in the benign group were always instructed to listen to the positive story across all story pairs, thus training them to attend to positive information and away from worry. In the control condition participants were asked to listen to the positive stories for half of the story pairs, and worry stories for the other half. The benign trained group demonstrated a more benign attention bias following training than the control group and also experienced few negative thought intrusions in a subsequent worry task, suggesting that attention bias has a role in maintaining worry. Longer-term attention training using multiple sessions of dot-probe training reduced attentional bias to threat words and importantly also led to reduced anxiety in people with GAD (64), indicating a causal role of attentional bias in maintaining anxiety in GAD. It should be noted, however, that while important in demonstrating the causal role for attentional bias, multi-session training methods designed to reduce attentional bias have sometimes failed to produce significant change in attention bias in other populations and therefore consequently anxiety (see for example a study in which internet-delivered training at home did not reduce social anxiety; 65). If CBM-A does not change the target process, then its role in maintaining anxiety or worry cannot be assessed. Further refinements to these methods are required to augment home based modification of attention bias. Despite this, evidence of an attentional bias to threat and its causal role in maintaining GAD has been supported, indicating that CBT interventions which facilitate a more benign attentional bias are warranted and may help optimize clinical outcome.

#### Representation of Threats in Generalized Verbal Form

Thoughts can occur in quasi-verbal form (as if talking to oneself), or imagery form (mental representations encompassing different sensory modalities). Evidence suggests that worry tends to occur predominantly in verbal form, with infrequent and brief images when they do occur (59–61). Furthermore, those with GAD have even briefer and fewer images than those without the disorder (61), in contrast to other anxiety disorders where prolonged negative imagery is common (66, 67).

Individuals with GAD sometimes report believing that worrying verbally is helpful in resolving their problems. This belief is misleading, however, as verbal worry has been found instead to increase subsequent negative thought intrusions (68) and prolong negative mood (69). One likely reason for this unhelpful effect is that verbally represented content in worry is typically over-general in nature, and easily moves from one negative topic to another, making positive resolution of specific problems difficult or impossible. Experiments have shown that intrusive thoughts following negative events are substantially more likely to persist if people are instructed to think about the event verbally (as in worry) rather than in the form of mental images (70). Similarly, Hirsch et al. (68) demonstrated that instructed practice in thinking about worry-related content in the form of mental images, which typically have a more specific and concrete focus, reduced the number of subsequent negative intrusive thoughts compared to engaging in worry in verbal form. Hirsch and Mathews (41) therefore propose that the primarily verbal nature of worry in GAD is particularly unhelpful and leads to greater capture of attention by threatening information (62), utilizes high levels of limited-capacity attention control resources (71) and promotes repeated bouts of worry by increasing the likelihood of subsequent negative thought intrusions (68, 72). Given this, CBT for GAD should encompass techniques that enable more imagery-based and concrete and specific thinking.

#### Defective Attentional Control

Another cognitive process proposed to underlie worry is impairment in attentional control (41). Attentional control is a limited capacity resource needed to intentionally ignore distracting information or to shift mental focus (73). Inducing active worry impairs attentional control resources (74). Unfortunately, attentional control is depleted in people with GAD (75, 76), with impairment particularly acute during worry (76, 77). Poor performance on attentional control tasks has also been found to predict subsequent development of GAD (78), further suggesting a causal role. Individuals with GAD may struggle to interrupt streams of worry and refocus onto other topics since worry occupies the same limited attentional control resources needed to refocus attention elsewhere. Furthermore, worry in verbal linguistic form may be particularly problematic for individuals who worry excessively. Leigh and Hirsch (71) found that high trait worriers performed poorly compared with low worriers on an attentional control task when worrying verbally, but not when they worried in imagery form. This suggests that the verbal thinking style typical of worry about negative events may be particularly unhelpful and lead to depleted attentional control, the resource needed to shift mental focus away from worry. Biased cognitive processes may combine with defective attentional control to perpetuate worry. Hirsch et al. (49) showed that cognitive bias modification of interpretation, which was designed to train high worriers to interpret ambiguous information more positively, not only facilitated a more benign interpretive bias and fewer negative thought intrusions, but also led to less impairment of attentional control during worry. Hence, interpretation bias may contribute to worry-specific attentional control problems, since more benign interpretations resulted in less pre-emption of attentional control resources by worry content. Given this, uncontrollable worry in GAD may be maintained in part by interpretive bias per se, but also by its on-going impact on attentional control (41). CBT for GAD needs to employ techniques that enable clients to utilize attentional control resources to focus on the task at hand and encourage them to shift away from worry (i.e. choose to deploy the attentional control resources they have on focusing externally). Furthermore, techniques which encourage imagery-based processing or facilitate benign interpretations are likely to also help clients deploy attentional control resources away from worry.

### Approaching CBT For GAD From A Cognitive Process Perspective

While traditional CBT (79) focuses on challenging negative thoughts, working at the cognitive content level with GAD can be less efficient, due to constantly shifting worry topics and multiple different perceived negative outcomes for any one worry. Hence, other CBT techniques that afford greater opportunity to change the dysfunctional cognitive processes that maintain worry are preferable. Borkovec’s CBT protocol (37, 38) forms the basis for our intervention since it is a gold-standard psychological treatment for GAD, and one of the CBT protocols recommended by the UK National Institute for Health and Care Excellence (18; other protocols include 22, 24, 26). Borkovec and Sharpless (80) outline how they selected and refined their CBT techniques to maximize potential change on key maintaining factors. Tom Borkovec comes from a behavioral perspective, and views behaviours as habits in much the same way as we view cognitive processes as thinking habits in our current approach. Borkovec and Sharpless (80) also highlight the need to focus on processes that appear particularly effective in reducing uncontrollable worry in GAD. Our work builds on this prior tradition of basing intervention selection for GAD on behavioral research, but draws more on recent relevant findings from cognitive research.

As discussed above, Hirsch and Mathews' (41) integrated model of pathological worry proposes that the three interacting cognitive processes discussed above—habitual cognitive-emotional processing biases towards threat (attention and interpretation), worry in generalized verbal-linguistic form, and depleted attentional control—combine to maintain pathological worry. Consequently, we selected therapeutic techniques and adapted existing interventions to maximize opportunities to target these key cognitive processes, either separately or in combination. Because each causal process can exert its effects on negative thought in different ways (41), achieving optimal improvements is likely to require targeting all of them in CBT. This may be achieved by facilitating more adaptive focus onto benign information (via intentional allocation of attentional control or more automated development of benign attention and interpretation biases), or engagement in more helpful thinking styles (concrete and specific imagery) evident in non-anxious populations. Furthermore, while Borkovec et al., (38) protocol was 16 sessions, routine clinical services—such as those in the UK NHS—aim to offer briefer interventions (e.g. 12 sessions) for anxiety disorders. Consequently, therapeutic techniques need to efficiently leverage change on multiple key cognitive processes.

### The Current Study

This paper presents an audit conducted in an NHS routine clinical service of an adaptation of Borkovec et al. (38) CBT protocol to focus on techniques that specifically target key cognitive processes outlined in Hirsch and Mathews (41) cognitive model. Up to 12 weekly sessions were offered rather than 16. The evaluation was conducted on consecutive GAD referrals to a routine clinical service in a UK NHS setting. Change in worry and anxiety were the primary outcomes, as the treatment focused on disorder-specific processes in GAD. Secondary outcomes were change in depression and functioning. Based on previous effectiveness studies of CBT for GAD and on promising evidence for targeting cognitive process variables, we hypothesized that using our revised protocol for CBT for GAD:

1. The intervention would yield significant pre-to-post treatment reduction in levels of pathological worry and anxiety.
2. The intervention would yield significant pre-to-post treatment reduction in levels of depression and functioning.
3. 50% of clients would achieve recovery on the PSWQ posttreatment (which would be in keeping with gold standard RCTs).

## Method

### Ethics Statement

All data were collected as part of routine service procedures/evaluation and thus did not require ethical approval. All patients and therapists were provided with information about how their clinical data was stored and used in routine service provision (81). Data were anonymized and processed in full accordance with the General Data Protection Regulation 2016.

### Participants

Participants had a primary GAD diagnosis and comprised 57 consecutive referrals for treatment for GAD at the Centre for Anxiety Disorders and Trauma (CADAT), South London and Maudsley NHS Foundation Trust. CADAT is a routine psychological care service with a specialist interest in the treatment of particular anxiety disorders (e.g. social anxiety disorder; panic disorder) but historically had not focused on GAD. All clients underwent a SCID (82) assessment for GAD at CADAT prior to treatment, and those with comorbidity identified that GAD was the primary problem that they wished to target. Inclusion criteria for the present evaluation included receiving at least one CBT session post-assessment, with clients attending a mean of 11.96 sessions including follow-up appointments (*SD*=2.91). Eighteen clients (31.58%) attended less than the typical and expected 12 treatment sessions, attending between 4 and 11 sessions. Nine of these clients (15.79% of total sample) attended 10 or 11 sessions (and were thus likely to have been given an adequate dose of treatment). Nine attended between 4 and 9 sessions. We performed intention-to-treat analyses including all clients’ data, with post-treatment scores on clinical measures derived from the final available session. Demographic characteristics of the client sample are reported in **Table 1**.

**Table 1:** Client Demographic Characteristics.

	Client Sample (*n*=57)
Age in years at start of treatment	Median = 33.00(IQR =13.50, range = 18–65)
**Gender**	
Female	75.44% (*n*=43)
Male	24.56% (*n*=14)
**Ethnicity**	
White	77.19% (*n*=44)
Mixed/Multiple Ethnicity	7.02% (*n*=4)
Black	5.26% (*n*=3)
Asian	1.75% (*n* =1)
Other	1.75% (*n* =1)
Undisclosed	7.02% (*n*=4)
**Employment Status**	
Full Time	56.14% (*n*=32)
Part Time	19.30% (*n*=11)
Student	10.53% (*n*=6)
Retired	5.26% (*n*=3)
Self-Employed	5.26% (*n*=3)
Unemployed	3.51% (*n*=2)
**Long-Term Physical Health Condition(data available for 52 clients)**	26.92% (*n*=14)
**Taking Psychotropic Medication(data available for 50 clients)**	46.00% (*n*=23)
**Previous Psychological Treatment(data available for 48 clients)**	
Yes—some form of previous treatment	72.92% (*n*=35)
No previous treatment	27.08% (*n*=13)

### Measures

Self-reported symptoms of worry in GAD were assessed with the 16-item Penn-State Worry Questionnaire (PSWQ; 33). Scores range from 0 to 8 on each item with caseness threshold total score ≥47 (31) and reliable change index ≥7 (33). The PSWQ has demonstrated good internal consistency α=.91–.95 and test-retest reliability *r*=.74–.93 (33) when measuring disorder-specific symptoms in adults with GAD.

Self-reported anxiety severity was assessed with the seven-item Generalized Anxiety Disorder-7 (GAD-7; 83): range = 0–21, caseness threshold ≥ 8, reliable change index ≥4. The GAD-7 exhibits good internal consistency, α=.92 and test-retest reliability, *r*(ICC)=.83 when measuring anxiety symptom severity in adults with GAD (83).

Self-reported depression severity was assessed with the nine-item Patient Health Questionnaire (PHQ-9; 84): range = 0–27, caseness threshold ≥10, reliable change index ≥6. The PHQ-9 exhibits good internal consistency, α=.89 (84) and test-retest reliability, *r*(ICC)=.84–.96 (85), when assessing the presence and severity of depressive symptoms in adults.

The impact of GAD on clients’ work, home and social functioning (functional impairment) was assessed with the five-item Work and Social Adjustment Scale (WSAS; 86). Scores range from 0 to 40, with <10 indicating minimal impairment, 10–20 indicating moderate impairment, and 20+ indicating severe impairment (86). The WSAS exhibits good internal consistency (α=.79–90; 86, 87) and test-retest reliability (*r* =.73; 86) as a measure of disorder-related functional impairment in adults with anxiety disorders.

### CBT For GAD Adapted To Target Key Worry-Related Cognitive Processes

Clients with GAD have numerous worry topics at any one time, and shift from topic-to-topic both within and between CBT sessions. Focusing the session can therefore be challenging and therapists may be drawn into “firefighting” individual worries, rather than seeing CBT as a means to develop more benign cognitive processes that can reduce worry in general. Our adaptations to Borkovec CBT interventions (37, 38) introduced or adapted techniques to maximize change on key cognitive process that maintain worry. While other techniques from the protocol are also used, below we discuss ones selected or adapted to target cognitive-emotional processing biases, or deployment of attentional control away from worry. The overarching aim of our adaptations to the protocol focus on helping clients overcome pre-potent cognitive biases and actively focus attention on the task at hand. To foster an understanding of the rationale for the interventions, we have found it useful to use more accessible terms to discuss the cognitive processes targeted in treatment and how more adapted processes can be viewed and developed during treatment. For example, as detailed more below, when talking to clients about worry and how hard it is to shift away worry it can be useful to refer to worry as a “mental magnet” and the need to refocus attentional control away from worry as shifting a “mental spotlight.” Cognitive biases are described to clients as “thinking habits” and that new more helpful thinking habits need to be developed via repeated practice. Developing these new thinking habits takes time and repetition, and this is explained to clients in terms of an analogy of repetitions of an exercise at a gym, which will lead to them developing new “mental muscles.” The selection and clinical adaptations were guided by the experimental data presented above, and how these techniques aim to target key mechanisms are described below. **Table 2** presents an overview of the targeted processes and the described techniques that target them.

**Table 2:** Worry-Relevant Cognitive Processes and Associated Techniques in CBT for GAD.

Cognitive process	CBT techniques that target the cognitive process
Attention	Formulation, worry history outcome, mental spotlight, worry free zone, worry timetabling, positive data log
Interpretation	Formulation, worry history outcome, positive data log, positive outcome imagery
Verbal thoughts	Formulation, worry history outcome, positive outcome imagery
Abstract generalized thinking	Formulation, worry history outcome, positive outcome imagery
Attention control	Formulation, mental spotlight, worry free zone, worry timetabling, positive data log

#### Formulation

Client and therapist work collaboratively to develop an idiosyncratic formulation based on a recent bout of worry. The formulation focuses on processes that trigger and maintain worry such as habits (cognitive-emotional processing biases) of attention and interpretation, as well as highlighting the thinking style being predominantly verbal and abstract in nature. By viewing cognitive biases as mental habits, clients can see that it will take time and effort to change their current tendency to worry, but that new habits can be developed to replace old ones, fostering hope of recovery. Furthermore, the role of depleted attentional control is also discussed in relation to the need to re-deploy a “mental-spotlight” onto the task at hand. The challenge for redeploying the “mental spotlight” is that the “mental magnet” of worry tends to keep the “mental spotlight” focused on worry. “Thinking habits” (i.e. cognitive processes that maintain worry) fuel the “mental magnet” keeping clients focused on their worry. In this way, the formulation highlights key cognitive processes of attention and interpretation biases, verbal abstract worry, and the difficulty of shifting attentional control away from worry and deliberately onto the task at hand.

**Figure 1:**
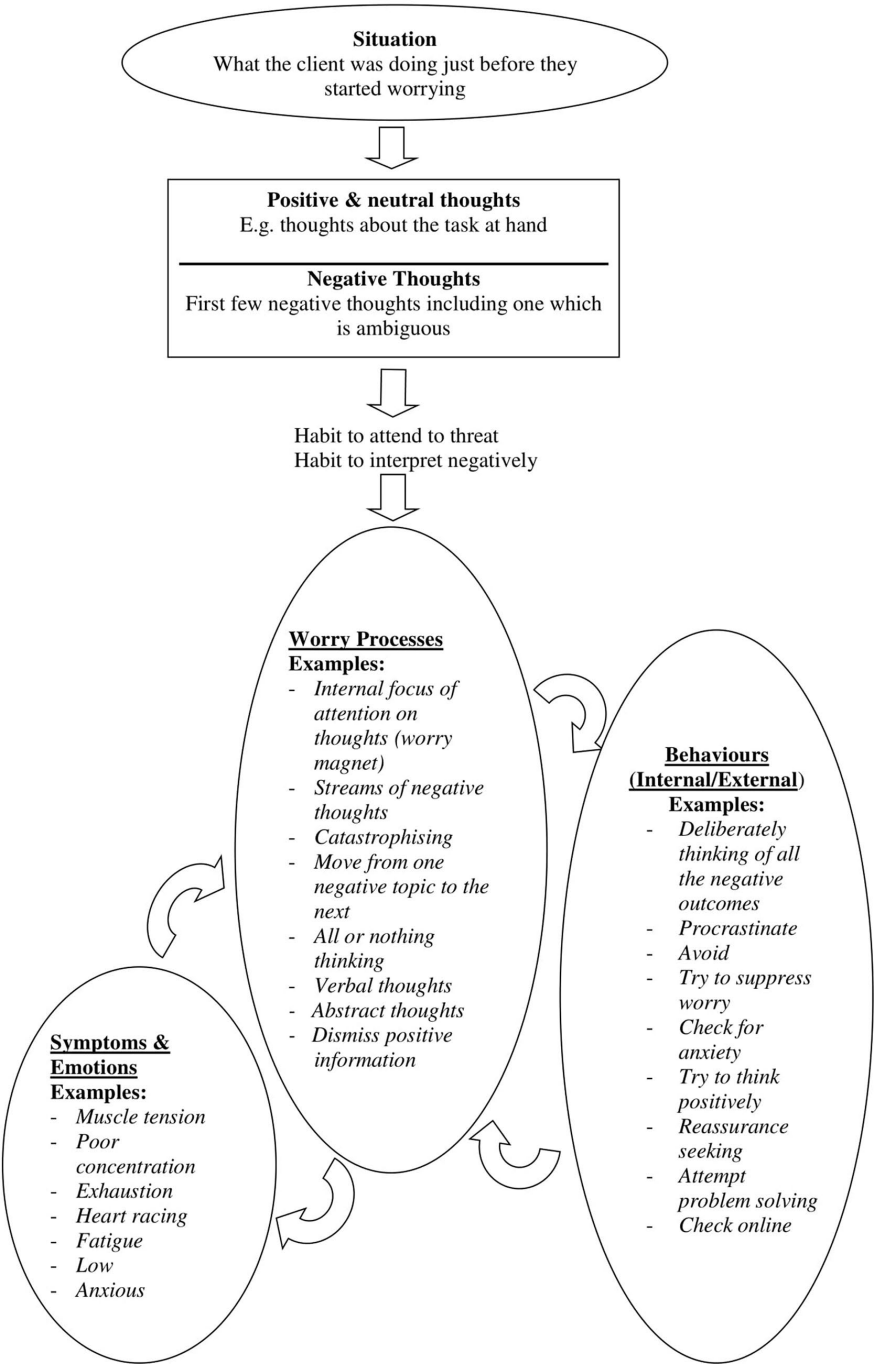
Typical formulation with examples of worry processes, behaviors, and symptoms.

Other information is also incorporated into the formulation. For example, when drawing out the processes that occur during worry, it can be useful to highlight any self-critical thinking. This often fuels worry and has the potential to undermine efforts to develop new CBT techniques, since if they are not deployed effectively on first attempt self-criticism often follows. This then increases emotional distress and promotes further worry. Consequently, having self-critical thinking style as part of the formulation is useful, and can be later countered by using a compassionate voice (88). The worry process itself also elicits physical symptoms of anxiety, lower mood and poor concentration. In turn, these symptoms can be focused on or interpreted negatively and can fuel more worry. Individuals will often try to respond behaviorally or by actively thinking in certain ways in an attempt to stop worry or deal with the situation. However, these behaviors can often lead back to worry or prove futile. The formulation forms the basis of the intervention, and provides a rationale for developing more helpful thinking habits (cognitive processes) and trying to shift focus away from worry and effectively onto the current task. Please refer to **Figure 1** for a typical formulation example.

#### Worry History Outcome Form

Individuals with GAD attend to thoughts around future threat and fail to attend to real benign outcomes for their worries (e.g. worrying about being late for work every day due to traffic, but not registering that they actually always arrive on time). The WHO form is used to record clients worry topics and evaluate whether or not negative outcomes actually occurred. It involves noting the worry topic and date on which the worry occurred, with each topic recorded only once until its outcome is known. In our adaptation of the techniques, clients are also asked to specify the concrete and specific feared outcome of the worry by briefly describing how a film director would set up the scene to show this outcome. This task promotes image-based thinking and ensures that the feared outcome is objective, concrete and specific and testable. Once the event has passed, clients rate whether the outcome was better or worse than expected (i.e. accuracy of the feared prediction) and how well they coped. Hence, the technique targets attention bias by requiring clients to attend to the real, typically positive, outcomes. This process of making an explicit assessment of the specified outcome may in turn provide an opportunity to counter any negative interpretations of the outcome, either at the time the rating is made, or later on reflection with the therapist when reviewing the WHO at subsequent sessions.

Over several sessions the number of worry topics accumulate. Therapists can address clients’ negative interpretations regarding their own performance or other’s responses generated by reviewing the outcomes; guided discovery highlighting perfectionist standards or viewing the situation less critically (e.g. as if it had happened to someone else) can be useful. After several sessions when situations are rated as better than expected, clients are asked to generate an image of the actual benign or positive outcome for thirty seconds. This is then repeated when any outcome is rated positively and provides practice in generating positive imagery.

After about six sessions of using the WHO form, the percentage of positive/benign outcomes (i.e. better than expected) is calculated for all events that have had an outcome. Borkovec et al. (89) cite that outcomes are better than anticipated 85% of the time. In our experience, rates of positive/benign outcomes very often exceed 95%, perhaps because the task was adapted to include a new column where the main concrete and specific feared outcome is explicitly noted on the form, and thus is more testable. For example, the topic may be “performance review” whereas the specific concrete feared outcome may be “John says I am performing poorly.” Personal data around positive outcomes are subsequently built on with a new technique later in treatment (positive outcome imagery—see below). The WHO form is thus used to target attention and interpretation biases, verbal thinking style, generalized and abstract thinking, and attentional control.

#### Mental Spotlight

Borkovec’s protocol (37, 38) involves clients trying to shift focus externally away from worry and onto the task at hand, which is conceptualized in our adaptation as shifting a “mental spotlight.” Unfortunately, shifting the mental spotlight onto the task at hand early in treatment is particularly difficult since worry utilizes attentional control, which is the very resource needed to shift attention away from worry. If the client manages to focus on the task at hand, they may find themselves drawn back to the “mental magnet” of worry due to cognitive biases. Additionally, stress and anxiety can further deplete attentional control resources already affected by GAD (76, 90). This makes it more difficult to implement CBT techniques for homework when clients feel most anxious or stressed, and yet this is the very time when they would benefit from CBT techniques the most. CBT homework should consequently be set up as repeated practice in developing new “mental muscles” (via more helpful focus of cognitive processes) to shift the mental spotlight. The aim is to practice the shift—not that people will always be able to be focus away from the worry—so any time the focus comes back on the worry or they are unable to shift, they can see it as another opportunity to practice the shift again. Informing clients that this can be challenging from the start will help them to remain engaged in CBT and see that being compassionate about this challenge, while still attempting to shift to the task at hand, will lead to longer term reductions in worry.

Introducing the concept of attentional control as a mental spotlight that can be difficult to shift and introducing CBT techniques as a way of developing new “mental muscles” that require numerous repetitions to develop helps to address potential barriers to progress with CBT. Over treatment, discussions about what clients will shift their mental spotlight to focus on—conceptualized as “hooks” to draw them in the task at hand—helps clients shift to the task at hand and enables them to remain engaged for longer periods of external focus by identifying particular aspects of a task to focus on. This demonstrates how CBT approaches, conceptualized through metaphors, combine to help clients shift focus away from worry by utilizing their attentional control.

#### Worry Free Zone

Worry free zones (WFZ) are introduced as the first opportunity to help clients practice focusing their mental spotlight externally onto the task at hand at times when they worry. WFZ were first introduced by Borkovec and Sharpless (80) but have been adapted to highlight the role of cognitive processes that maintain worry. During the first week, WFZ are short (e.g. 5 min) periods of time where clients try to focus away from worry externally onto the task at hand. The zones can be a specified task (e.g. making a cup of tea), place (e.g. bathroom) or time period (e.g. from waking until going downstairs). Clients should be prepared to expect that worry will naturally come back into their mind and to be compassionate with themselves when this happens and re-focus back on the current task. Later, the duration and number of worry-free zones can be increased. WFZ target attention control redeployment actively onto the clients’ current task, helping to override prepotent cognitive biases that will focus clients back onto worry. WFZ may also promote attention bias to benign information.

#### Worry Timetabling

Once clients can shift focus away from worry during WFZ, they could move on to worry timetabling. Worry timetabling requires the client to postpone worries until a specified time later in the day (e.g. 15 min at 5pm) when they catch themselves worrying, and then re-focus their mental spotlight onto the task at hand. Again, clients may need to be reminded of the importance of using their compassionate voice when they notice the worry returning to their mind. Initially worry may return very quickly, but with practice will return less often and with longer worry-free intervals. If clients forget to use the worry period, then they are asked to timetable any subsequent worries to the next day’s worry period.

During the first week, the worry period is just left as a time when clients can worry. However, during the following session the therapist will enquire about how the client found the experience of postponing worry, whether the worry returned immediately, and if they persevered with the technique, whether the time between worry bouts about that topic grew longer. The therapist should also enquire about when it was harder and easier to postpone worry to help tailor techniques to facilitate greater ability to shift from worry and remain focused on the task at hand. Clients may subsequently choose to not worry in the worry period, but think about worries in more objective ways, or not at all. The worry timetabling technique utilizes attentional control, which is deployed onto the task at hand, and consequently also helps develop a new attention bias to benign information since most tasks are benign in nature.

#### Positive Data Log

While not part of Borkovec’s protocol (37, 38), keeping a positive data log can help to develop more adaptive thinking habits. Padesky (91) introduced the technique to help develop more adaptive core beliefs by having clients attend to and note down evidence in day to day life that is in keeping with their new alternative (adaptive) core belief. While the current protocol does not focus on core beliefs per se, worry is often driven by a sense that the individual is “not good enough” and so working with the client to collect evidence that they are “OK” in terms of what they do and how others respond to them is useful. The positive data log involves writing down evidence in day-to-day-life that they are OK. Clients can aim to write down a few pieces of evidence on their log each day. It also has the function of getting the client to be a “detective for positive outcomes” and thus helps develop a more benign attentional bias. Furthermore, when identifying potential information for the positive data log, this may also provide an opportunity to generate positive interpretations of ambiguous events in day-to-day life.

#### Positive Outcome Imagery

Imagery techniques can be used when people are worrying, and the outcome is unknown. Clients identify a current worry topic and specify a concrete and specific feared outcome and rate the percentage likelihood that this outcome will occur. Given that their WHO collation conducted earlier in treatment will indicate that worry topics very often have benign or positive resolutions (85% of the time; 89), clients are requested to brainstorm different ways that this situation could turn out well. This task develops a new habit to generate multiple positive outcomes, rather than multiple negative outcomes characteristic of worry. Clients are then requested to select an outcome or combination of outcomes to think about further in a positive, concrete way. Clients are asked to set-up the scene as if they were a film director, making the outcome concrete and specific. Clients then close their eyes and generate a vivid image of the scenario unfolding, tuning into the different sensory modalities for 2 min. Finally, they re-rate the likelihood the feared outcome would happen. This technique promotes attention to positive information, positive interpretations and concrete and specific positive outcome imagery of future worries.

### Procedure

All clients completed at least one CBT for GAD treatment session with a therapist accredited with the British Association for Behavioural and Cognitive Psychotherapies, following an initial assessment. Clients completed clinical measures just prior to assessment (pre-treatment) and at end of treatment just prior to the final clinical session (post-treatment). Two clients did not complete pre-treatment WSAS, so were missing scores at this time point.

## Results

Paired-samples t-tests were conducted to compare mean pre-treatment and post-treatment scores for the PSWQ, GAD-7, PHQ-9, and WSAS. Effect sizes of the mean difference for each measure were estimated using Cohen’s *d* with Morris and DeShon (92) Equation 8 applied to correct for dependence between means. Significant differences were found between the pre-treatment and post-treatment questionnaire for all measures, with large effects indicated for the PSWQ, GAD-7 and PHQ-9, and moderate effects for the WSAS (small ≥.20, moderate ≥.50, large ≥.80; 93). **Table 3** presents the findings.

**Table 3:** Mean Change in Clinical Outcome Measures Pre- and Post-Treatment.

Measure	Cases with paired scores (*n*)	*M*_pre_ (*SD*_pre_)	*M*_post_ (*SD*_post_)	*df*	*t*	Cohen’s *d*
PSWQ	57	70.72 (6.97)	47.56 (10.84)	56	14.91**	2.54
GAD-7	57	14.16 (5.32)	5.05 (4.06)	56	13.11**	1.74
PHQ-9	57	11.32 (6.59)	5.12 (4.85)	56	6.80**	.90
WSAS	55	15.20 (8.16)	9.49 (7.13)	54	4.47**	.74

**p≤.001.

PSWQ, Penn State Worry Questionnaire, GAD-7, Generalized Anxiety Disorder -7; PHQ-9, Patient Health Questionnaire; WSAS, Work and Social Adjustment Scale.

Reliable change rates were computed for the PSWQ, GAD-7, and PHQ-9 to assess the clinical significance of change across treatment. Cases demonstrated reliable improvement if their scores decreased between pre-treatment and post-treatment beyond the reliable change index for the given measure (i.e. PSWQ ≥ 7, GAD-7 ≥4, PHQ-9 ≥6; 94). Likewise, cases demonstrated reliable deterioration if their scores increased beyond the reliable change index. No reliable change was indicated if scores changed less than the reliable change index in either direction. The majority of cases demonstrated reliable improvement on the PSWQ and the GAD7, and no reliable change on the PHQ9. Relatively low rates of reliable change on depression (PHQ-9) were probably driven by low pre-treatment depression severity, with only 36 clients exceeding the PHQ-9 caseness threshold for clinically significant symptoms of depression pre-treatment (PHQ-9 ≥10). Of the 36 clients who were above clinical cut off pre-treatment, 72.22% (*n*=26) demonstrated reliable improvement and 27.78% (*n*=10) demonstrated no reliable change. Rates of reliable deterioration for all measures were very low. No reliable change index was available for the WSAS. **Table 4** presents the reliable change findings.

**Table 4:** Reliable Change Rates on Outcome Measures.

Measure	*n* cases with paired scores	Reliable Deterioration % (*n*)	No Reliable Change % (*n*)	Reliable Improvement % (*n*)
PSWQ	57	1.75 (1)	3.51 (2)	94.74 (54)
GAD-7	57	1.75 (1)	15.79 (9)	82.46 (47)
PHQ-9	57	1.75 (1)	50.88 (29)	47.37 (27)

PSWQ, Penn State Worry Questionnaire, GAD-7, Generalized Anxiety Disorder -7; PHQ-9, Patient Health Questionnaire.

Recovery rates were also computed based on the clinical outcome measures. Cases were considered recovered if they were above the caseness threshold for the measure pre-treatment (i.e. PSWQ ≥ 47, GAD-7 ≥8, PHQ-9 ≥10) and decreased below the threshold post-treatment (94). In keeping with post-treatment recovery rates from gold-standard trials (i.e.46%; 31), over 50% of all cases recovered on the PSWQ. Recovery rates were strong for the GAD-7 and PHQ-9, and substantially exceeded the minimum 50% recovery rate threshold on generic measures stipulated in NHS primary care psychology service guidelines (94). No recovery index was available for the WSAS. **Table 5** presents the findings on recovery.

**Table 5:** Recovery Rates on Outcomes Measures.

Measure	Cases above Threshold pre-treatment *n*	Recovered % *(n*)	Not Recovered % *(n*)
PSWQ	57	52.63 (30)	47.37 (27)
GAD-7	47	74.47 (35)	25.53 (12)
PHQ-9	36	77.78 (28)	22.22 (8)

PSWQ, Penn State Worry Questionnaire, GAD-7, Generalized Anxiety Disorder -7; PHQ-9, Patient Health Questionnaire.

## Discussion

GAD has uncontrollable worry at its core. CBT is a first-line treatment for GAD, so targeting cognitive processes that maintain worry should be a key focus. The current service audit aimed to investigate the effectiveness of CBT for GAD that was adapted to maximize potential impact on key processes which maintain worry, based on an evidence-based cognitive-process model of pathological worry. As predicted, clients demonstrated significant pre-to-posttreatment reduction in worry, general anxiety, and depressive symptoms with large effects (*d*=.90–2.54), and in functional impairment with moderate effects (*d* =.74). Reliable improvement was notably high for anxiety (82%) and worry (95%). Recovery determined by cut off scores was 74% for anxiety and 78% for depression. Also, as predicted, over 50% of cases achieved recovery on worry using the PSWQ (52.6%), in keeping with gold standard clinical trials. These findings demonstrate that formulating with cognitive processes in mind and adapting key techniques to address cognitive processes enables clients to benefit from CBT.

This audit provides evidence of significant treatment effects on both disorder-specific (i.e. pathological worry) and generic (i.e. general anxiety, mood, and functional impairment) clinical outcome measures in line with pre-to-posttreatment effects of efficacy trials of CBT for GAD (23, 29, 31). Notably, effect sizes exceeded previous estimates of effectiveness in routine care for measures of worry, which in our service was *d*=2.54 compared to *d*= 0.61- 0.96 (95, 96), anxiety (our service *d*=1.74 compared to *d*=0.92, 36; and *d* =1.13, 97), and depression (our service *d*= 0.90 in keeping with *d* =.89, 36). These strong outcomes were obtained with 12 sessions, which was briefer treatment than the 16-session Borkovec et al. (38) protocol and many previous effectiveness studies in routine care (12–25 session protocols: 98–100). These findings indicate that tailoring interventions to prioritize potential change on key cognitive processes that maintain GAD can provide helpful and efficient treatment.

The current study also had the benefit of assessing rates of reliable change—or change beyond the measurement error of the given clinical outcome measure—which were promisingly high for pathological worry (95%) and general anxiety (82%). The lower rate of reliable change for depression symptoms (47%) is potentially explained by relatively low pre-treatment depression severity, with a mean pre-treatment PHQ-9 score just exceeding the caseness threshold (total score ≥10), and with 21 clients not meeting depression caseness criteria at baseline. The majority (72.22%) of clients with clinically significant pre-treatment depression scores demonstrated reliable improvement. Recovery rates also exceeded the NHS service targets of 50% for all measures, with the 52% recovery rate observed for the PSWQ in this evaluation in line with meta-analytic posttreatment estimates for gold standard RCTs (46%, 32). Unfortunately, recovery is rarely measured using disorder-specific scales in routine care, as highlighted by Clark (101). The current evaluation also outperformed previous routine care studies in regard to recovery rates for general anxiety symptoms (74% in the current study versus 35%, 98; 43%, 97). Given that effect sizes and recovery rates in the current evaluation were comparable to efficacy trials and exceeded previous routine care studies that focused on GAD in for several relevant clinical outcomes, findings indicate that adapting CBT protocols in line with the emerging evidence-base around underlying processes in GAD may strengthen the outcome of relatively brief treatment in routine care. While results are encouraging, application of the cognitive-process model with CBT may require further refinement to further bolster clinical outcomes, particularly recovery rates on disorder-specific measures of pathological worry such as the PSWQ. That said, the rates are in keeping with gold standard trials of CBT for GAD, and thus this is also an issue for the field more generally.

While these adaptations were made to the Borkovec protocol (37, 38) and built on Borkovec and Sharpless, (80) focus on selecting techniques which target key behavioral targets, similar adaptations could potentially be used to select and refine key interventions used in other CBT treatment protocols for GAD. Furthermore, the beneficial impact of CBT evidenced in the current audit is attributable to the overall CBT package and we cannot determine what impact our refinements have had. Furthermore, we do not wish to suggest that Borkovec’s original techniques, which were designed target behavioral processes, were not critical ingredients for the encouraging clinical outcomes we observed. Indeed, due to this being an audit of routine care, we do not assess mechanisms of change in the current study, which is an important focus for future research.

Recent research has demonstrated that multi-session cognitive bias modification (CBM) for interpretations reduces anxiety and worry in individuals with GAD (51). Examining the feasibility and effectiveness of incorporating these methods into homework for CBT may facilitate greater and more rapid reductions in worry. Furthermore, CBM for interpretation that is enhanced with prolonged imagery and self-generation of outcomes may be particularly helpful in this regard, in a similar manner to using interventions in CBT that target multiple cognitive biases simultaneously. 102) has shown that interpretation training enhanced in this manner augments impact on interpretation bias and could be a promising form of CBM to incorporate into cognitive-process-focused CBT for GAD. Further investigation of clinical outcomes for CBT for GAD incorporating these CBM with imagery and self-generation of outcome is indicated, particularly to determine if this could make face to face CBT briefer.

While the findings from the present evaluation provide encouraging support for CBT for GAD informed by the cognitive-process model of pathological worry, they are subject to several limitations inherent to the naturalistic design. While outcomes were similar to those seen in previous randomized control trials, the present evaluation did not include a control condition. As data were collected as part of routine service procedures, results are not generalizable beyond the specific service context. The clinicians in the present evaluation were also highly trained and experienced in delivering CBT for anxiety disorders, which may preclude representativeness to other routine service settings. Given that the evaluation was based on routine clinical practice, the number of sessions was adapted to clients’ needs and constrained by service demands rather than controlled. Only client self-rated outcomes measures were routinely used in the service, and further investigation of clinical change based on independent clinician-rated measures is warranted, given that self-rated measures may exhibit larger effect sizes for pre-to-posttreatment change in anxiety disorders (27). Additionally, while the screening procedures in the present service ensured that DSM V diagnosis was recorded for GAD for all clients, there was insufficient information available to accurately report age of onset, duration of disorder, and comorbidity. The client sample was also majority female (75%), potentially affecting generalizability of the findings. Due to the preliminary nature of this evaluation and data availability, medication status and other potentially relevant clinical and demographic factors were not controlled for in the analyses. As the evaluation was conducted in routine care, it was not feasible to include follow-up of clients. This is a priority of future research, and efficacy trials indicate that effect sizes and recovery rates may be maintained or increase long-term (31, 97, 98). Additionally, it was not feasible to measure therapists’ adherence to the protocol and use of each therapeutic technique in the present evaluation. To build upon the encouraging findings of the present evaluation, a full randomized control trial of CBT for GAD informed by the cognitive-process model of pathological worry is warranted in the future. Future trials could enable the important assessment of change in key cognitive processes, assessed using appropriate experimental methods, prior to and following treatment, to determine whether these are ameliorated as desired via CBT and whether these processes mediate longer term reductions in worry and anxiety. Further, if cognitive process-informed CBT for GAD continues to demonstrate promising outcomes in adult samples, adapting CBT for GAD in children and young people based on corresponding evidence of relevant cognitive processes in this population may be warranted.

Conclusion: Techniques that maximize the impact of interventions on key cognitive processes that maintain worry can lead to effective treatment. Formal evaluation of CBT for GAD guided by a cognitive process view of GAD in the form of a full randomized control trial is consequently indicated to continue to strengthen client outcome for this common and debilitating condition.

## Data Availability Statement

The datasets generated for this study will not be made publicly available as they comprise audit of a clinical service.

## Ethics Statement

All data were collected as part of routine service procedures/evaluation and thus did not require ethical approval. All patients and therapists were provided with information about how their clinical data was stored and used in routine service provision (South London and Maudsley NHS Foundation Trust, 2011). Data were anonymised and processed in full accordance with the General Data Protection Regulation 2016.

## Author Contributions

CH, SL, and NG contributed conception and design of the study. SB created the database and performed the statistical analysis. CH and SB wrote sections of the manuscript. All authors contributed to manuscript revision, read and approved the submitted version.

## Funding

This research did not receive any specific grant from funding agencies in the public, commercial, or not-for-profit sectors. CH receives salary support from the National Institute for Health Research (NIHR), Mental Health Biomedical Research Centre at South London and Maudsley NHS Foundation Trust and King’s College London. The views expressed in this article are those of the author(s) and not necessarily those of King’s College London, the NIHR, or the Department of Health.

## Conflict of Interest

The authors declare that the research was conducted in the absence of any commercial or financial relationships that could be construed as a potential conflict of interest.

The reviewer ZM declared a shared affiliation, with no collaboration, with several of the authors CH, SB, and SL to the handling editor.
